# Preventing Cervical Cancer in the United States: Barriers and Resolutions for HPV Vaccination

**DOI:** 10.3389/fonc.2016.00019

**Published:** 2016-02-01

**Authors:** Anna Louise Beavis, Kimberly L. Levinson

**Affiliations:** ^1^The Kelly Gynecologic Oncology Service, Department of Gynecology and Obstetrics, Johns Hopkins Hospital, Baltimore, MD, USA

**Keywords:** HPV, vaccination, cervical cancer, disparities, health policy

## Abstract

Human papillomavirus (HPV) vaccination rates for preadolescent and adolescent girls in the United States are far behind those of other developed nations. These rates differ substantially by region and state, socioeconomic status, and insurance status. In parents and young women, a lack of awareness and a misperception of the risk of this vaccine drive low vaccination rates. In physicians, lack of comfort with discussion of sexuality and the perception that the vaccine should be delayed to a later age contribute to low vaccination rates. Patient- and physician-targeted educational campaigns, systems-based interventions, and school-based vaccine clinics offer a variety of ways to address the barriers to HPV vaccination. A diverse and culturally appropriate approach to promoting vaccine uptake has the potential to significantly improve vaccination rates in order to reach the Healthy People 2020 goal of over 80% vaccination in adolescent girls. This article reviews the disparities in HPV vaccination rates in girls in the United States, the influences of patients’, physicians’, and parents’ attitudes on vaccine uptake, and the proposed interventions that may help the United States reach its goal for vaccine coverage.

## Introduction

Nearly 13,000 American women will be diagnosed with cervical cancer in 2015, and over 4,000 women will die from cervical cancer (1). With the introduction of regular Papanicolaou (Pap) smear screening in the 1950s, cervical cancer incidence rates dropped over 80% (2). While this represents a huge public health success, there is potential for even greater impact on this disease with the human papillomavirus (HPV) vaccination. HPV is the known necessary cause for cervical cancer, and in 2006, the first vaccine targeting HPV was approved in the United States for prevention of both cervical cancer and genital warts. This four-valent vaccine (trade name Gardasil, Merck & Co., Inc.) is active against HPV genotypes 6, 11, 16, and 18, which are responsible for approximately 66% of cervical cancers and 90% of genital warts. It is administered as three injections over 6 months (3). In 2006, the Advisory Committee on Immunization Practices (ACIP) recommended HPV vaccination as a routine vaccine for girls aged 11–12 and approved it for all women up to the age of 26 (4). In 2009, a bivalent vaccine, targeting oncogenic HPV genotypes 16 and 18 was also approved (trade name Cervarix, GalaxoSmithKline) (4), which was found to be similarly efficacious against cervical cancers caused by these HPV genotypes (3). More recently, in December 2014, the Food and Drug Administration (FDA) approved a nine-valent vaccine (trade name Gardasil 9, Merck & Co., Inc.) that covers five additional HPV genotypes (31, 33, 45, 52, and 58), which are responsible for an additional 15–20% of cervical cancer cases (5). Soon after, in February 2015, the ACIP incorporated the nine-valent vaccine into its recommendations for routine recommendation as an alternative to the four- and bivalent HPV vaccines (6). Phase III trials of the newest vaccine show over 95% efficacy against the additional HPV genotypes (5); therefore, vaccinating the next generation of young women has the potential to prevent almost 90% of cervical cancer cases.

The development of a vaccine against HPV was a major breakthrough in science, but its potential public health success heavily depends upon the acceptance and uptake in any given population. Compared to other developed nations, the United States has been slow to vaccinate. In the first year after the vaccine was approved, only 11.6% of American girls aged 13–17 received at least one dose (7), in contrast to over 80% of girls aged 13–17 who initiated vaccination in Australia (8). One of the key differences between these two countries is the way in which Australia approached the public health need: a successful nationwide, school-based, government-funded HPV vaccination campaign was launched in 2007 (8). While it is too early to show reductions in cervical cancer rates in those vaccinated, the prevalence of genital warts in Australian women under 21 years of age has dropped over 90% in the last 5 years, compared to no change in the rates for women over the age of 30, who did not receive vaccine (9).

Since 2007, vaccine uptake rates have improved in the United States. However, they are still well below goal. Two recent national surveys estimated that between 34 and 60% of eligible girls of ages 11–26 years have received at least one dose of the vaccine (i.e., vaccine uptake), and fewer than half complete the entire three dose regimen (i.e., vaccine completion) (7, 10). Healthy People 2020 has set a goal to reach 80% vaccine completion in girls aged 13–15 by the year 2020 (11). In order to achieve this, vaccination rates will need to more than double in the next 5 years. Understanding the country-specific factors behind the low vaccination rates will help inform interventions to improve uptake in the United States.

In this review, we will evaluate the recent research in disparities in vaccine uptake in the United States, examine key stakeholders’ attitudes toward the vaccine, and consider potential interventions that may help improve vaccination uptake rates in the United States. While this vaccine has been approved and is recommended for boys as well (4), this review will focus on the available research for vaccination in girls.

## Notable Disparities in HPV Vaccination

In the United States, cervical cancer disproportionately affects women of low socioeconomic status, minority populations, and those with limited access to the health-care system (12, 13). The racial disparities may be even more pronounced than previously thought (14). Differences in HPV vaccination initially paralleled these same racial and socioeconomic disparities, but recent data suggest that racial and socioeconomic disparities have decreased significantly (15). However, differences in vaccine uptake are still pervasive by region, insurance status, and sexual orientation (16–19).

### Regional Differences

**Figure 1:**
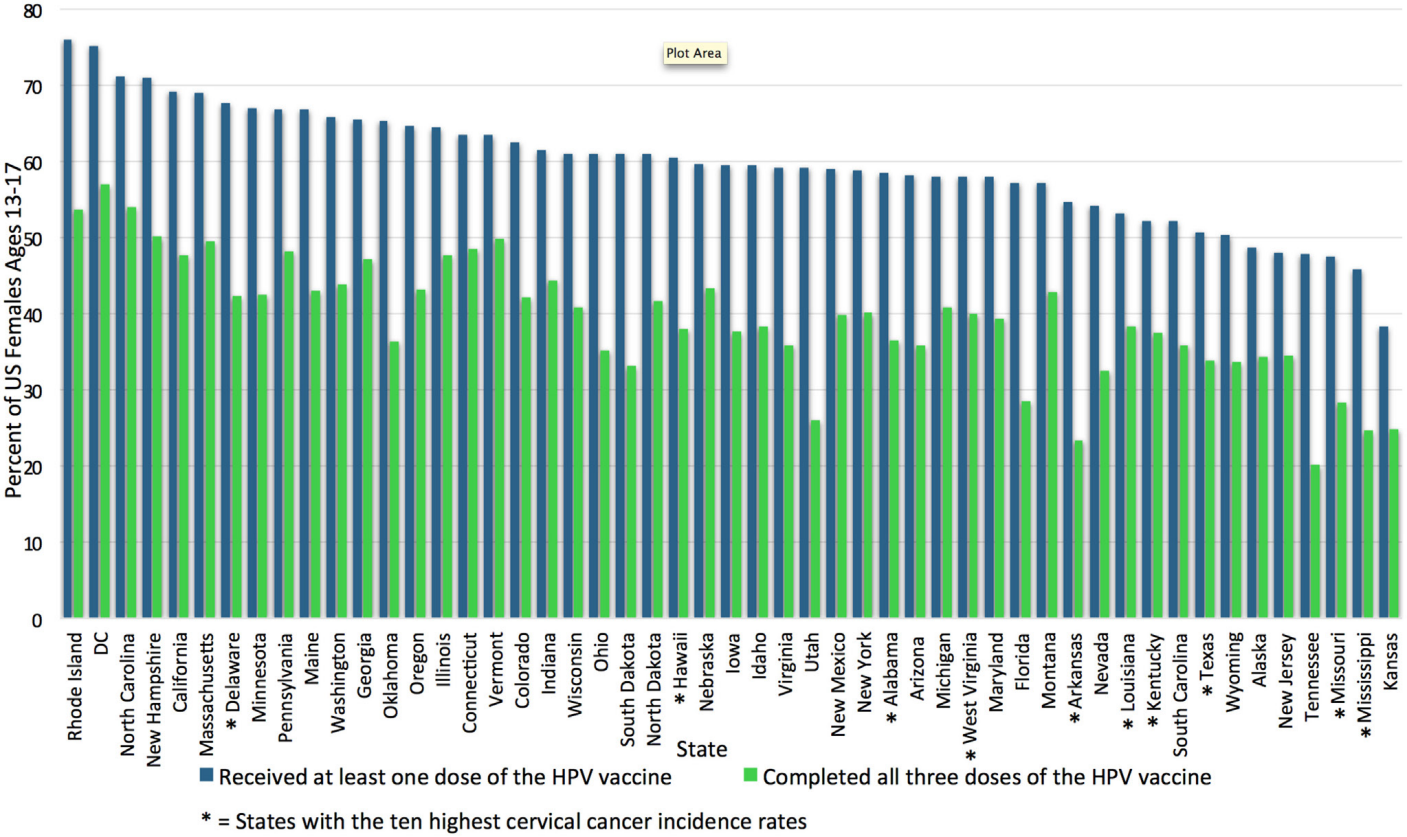
**Uptake and completion rates of HPV vaccination by state**. This graph shows the percentage of female adolescents aged 13–17 who initiated and completed the HPV vaccination series in the United States in 2014, by state. HPV vaccination uptake ranges from 76% in Rhode Island to 38.3% in Kansas (15). The 10 states with the highest cervical cancer incidence rates according to the CDC in 2012 are marked with *; most are among those with the lowest HPV vaccination rates (24). HPV vaccination rates adapted from the NIS-Teen survey data reported in Reagan-Steiner et al., (15). DC, District of Columbia; HPV, human papillomavirus.

Human papillomavirus vaccination rates vary widely by state. In 2009, HPV vaccination initiation was highest for the Northeast and Midwest regions of the United States and lowest in the Southeast (20). Additionally, family physicians located in the South less routinely offer the vaccine when compared to family physicians in the Northeast, Midwest, and West regions of the United States. These differences were not seen among pediatricians, who have high rates of vaccine delivery nationwide. This may be due to pediatricians’ emphasis on immunization in their education and scope of practice, as pediatricians were also more likely to participate in the Vaccines for Children Program, a federally funded immunization program. Family physicians are also historically slower to incorporate any vaccine recommendation compared to pediatricians (21). The National Immunization Survey-Teen (NIS-Teen), an annual survey used by the Center for Disease Control (CDC) to monitor vaccination coverage in adolescents, surveyed over 20,000 adolescents in all 50 states and the District of Colombia (DC) in 2014 and found that Kansas has the lowest state-level HPV vaccine uptake rate: only 38.3% of girls aged 13–17 had initiated vaccination, while Rhode Island has the highest: 76% of girls aged 13–17 had initiated vaccination (10). Unfortunately, the states with the highest cervical cancer rates also have some of the lowest HPV vaccination rates (22). Figure 1 demonstrates the vast state-wide differences in HPV vaccination initiation and completion in girls aged 13–17 during 2014 according to the NIS-Teen survey data. These regional differences may reflect how much each state’s government has chosen to encourage the vaccination. Washington, DC, USA, for example, puts resources into both educational interventions and mandates the vaccine; likely as a result, the percentage of girls receiving at least one dose rose from 38.7% in 2008 to 75.2% in 2014, second only to Rhode Island (15, 23).

### Ethnic/Racial Disparities

Minority populations – black women and Hispanic women specifically – are more likely to be diagnosed with cervical cancer (14). HPV also appears to be more prevalent in black women compared to all other racial and ethnic groups in the United States (25). In the first several years after the HPV vaccine was made available, studies reported that young black women were less likely to initiate vaccination, and if they did initiate vaccination, they were less likely to complete the vaccination series than other racial groups (16, 26, 27). Fisher et al. performed a meta-analysis of the available data from vaccine inception to March 2012 and confirmed this finding [OR 0.89 comparing vaccine initiation in non-Hispanic black women to non-Hispanic white women, 95% confidence interval (CI): 0.82–0.97] (19).

However, in the more recent literature, estimates of vaccine uptake by racial category demonstrate positive change and may reflect a rapidly changing landscape of vaccine acceptability and accessibility. Data from the 2014 NIS-Teen survey show that more non-Hispanic black adolescents received at least one dose of the HPV vaccine than their white counterparts (66.4 vs. 56.2%, *p* < 0.05). Compared to NIS-Teen data from 2008, where vaccine uptake rates of both races was around 35%, these rates show improvements in uptake for both groups over time (23). However, rates of receipt of all three doses remain low for both groups (39 vs. 37.5% non-Hispanic black vs. non-Hispanic white women, *p*-value not given) (15).

It is unclear whether vaccination rates among Hispanic and Asian women differ from rates in non-Hispanic white women. A systematic review of the studies comparing vaccine rates among these groups was inconclusive (19). In the 2014 NIS-Teen survey, Hispanic adolescents were both more likely to initiate vaccination compared to non-Hispanic whites (66.3 vs. 56.1%, *p* < 0.05) and more likely to have received three doses (46.9 vs. 37.5%, *p* < 0.05) (15). While these data are encouraging and demonstrate a closing gap in racial and ethnic disparities, the fact that fewer than half of all groups complete the series indicates that there is significant room for improvement (21).

### Sexual Orientation

While the HPV virus is prevalent in women who have sex with women (28), this group of women is less likely to report appropriate Pap screening (29). Gay, lesbian, and bisexual women or “sexual minority adults” are also more likely to smoke than their “non-minority” counterparts (30). Smoking increases the chances of HPV-related cervical changes, persistence of HPV (31), and progression from cervical dysplasia to invasive cancer (32). Therefore, regardless of sexual orientation, national screening and vaccination recommendations apply and may be even more important. However, an analysis of the 2006–2010 National Survey of Family Growth (NSFG) found that lesbian women are less likely to have initiated HPV vaccination than their heterosexual counterparts (8.5 vs. 28.5%, *p* = 0.007). In this study, there were no differences between bisexual women and heterosexual women in vaccine initiation. These percentages should be interpreted with caution, as the sample sizes for these subgroups of women were small (*n* = 62 for lesbian women and *n* = 235 for bisexual women); however, the differences among these different groups remain concerning (17). A national online survey targeted at the lesbian, gay, and bisexual community, which included 543 gay and bisexual women aged 18–26 found more encouraging results: 45% reportedly had initiated the HPV vaccine (18). While the NSFG survey may be an underestimation due to its small numbers, the online survey may be an overestimation as those volunteering to participate in online health surveys may be more likely to have received health care and vaccination, and they failed to differentiate between lesbian and bisexual women. Due to the small number of other studies addressing vaccine uptake in gay and bisexual women, the true percentage of vaccine initiators in this group is not known. Given the available data, however, vaccine uptake among sexual minority adults appears lower than the average of 60% for all American female adolescents aged 13–17 (10).

### Socioeconomic and Insurance Status

In the United States, cost is frequently cited as a barrier to HPV vaccination initiation and completion (18, 21, 27, 33). Historically, women of lower socioeconomic status were less likely to initiate and complete the HPV vaccine series (16, 19, 27). In 2008, pediatricians and family practice physicians reported that financial concerns were the most frequently cited reasons for not vaccinating (21). Much of this, however, may be explained by insurance status. Women with any insurance in the United States are much more likely to have been counseled about the HPV vaccine, are more likely to intend to get vaccinated, and are more likely to have initiated vaccination than uninsured women (16, 19, 33, 34). A meta-analysis demonstrated that lack of health insurance, rather than income itself, was one of the most important factors associated with failure to initiate HPV vaccination (19). This finding is very important from a public health perspective, as this plays a critical role in determining strategies that could improve vaccination initiation and completion.

The passage of the Affordable Care Act (ACA) in 2010 is one major public health change that is expected to improve HPV vaccination. Under the ACA, any person with insurance can receive the HPV vaccine without any additional cost sharing (i.e., they do not have to pay a copay) when they go to an in-network provider (35). If the cost of vaccination is truly a pivotal barrier, a rise in vaccine uptake should be seen after the passage of the ACA.

## Stakeholders’ Knowledge and Attitudes toward HPV Vaccination

An understanding of the various stakeholders’ knowledge and attitudes about the HPV vaccine is critical to understand why vaccination has not been more widely accepted and to help inform strategies to improve its uptake.

### Parents’ Knowledge and Attitudes

Generally, parents’ knowledge of HPV in the United States is poor. In a subset of the National Health Interview Survey 2010, 4 years after the four-valent vaccine was approved, only 63% of 5,735 parents of children aged 8–17 had even heard of the HPV vaccine (36). Another survey 3 years later failed to show any meaningful improvement: 68% of American adults had heard of the HPV vaccine (37). Parents who lack knowledge frequently cite concerns about side effects and vaccine efficacy as reasons not to vaccinate their daughters (21, 38, 39). Some of this lack of knowledge may be related to lack of education, which may result in failure to vaccinate: mothers with lower education level are less likely to have their daughters vaccinated than mothers with higher education level (26).

Parents’ attitudes toward their children and the vaccine also influence vaccination rates. Parents who perceive their daughter to be at low risk of sexual activity often fail to vaccinate their daughters (39). Parents are more likely to refuse or delay vaccination for girls who are 11–12 years old than for girls who are 13–15 years old, and concerns about sexual activity, including the unfounded concern that receipt of the vaccine will result in risky sexual behavior, are associated with these delays (21, 40). Open discussion with the physician may help to alleviate these fears and change parents’ attitudes regarding vaccination, as parents who did not feel they could discuss their concerns with their physician were more likely to not vaccinate their child (38). Additionally, a mother’s own health practices influence her decisions about her daughter: mothers with more exposure to a primary care physician who regularly receive preventive care (e.g., Pap smears, mammograms) are more likely to agree to vaccinate their daughters, perhaps reflecting the value they place in preventive medicine (41, 42).

### Patients’ Preferences

While over 80% of young women (ages 15–25) have heard of the HPV vaccine (17), young women express similar concerns as their parents with regards to adverse effects and efficacy (18, 43). On the one hand, many girls and women who do not intend to get vaccinated cite a perceived low risk for HPV as their reason against vaccination. On the other hand, young women who report that they do intend to get vaccinated are more likely to have already had sex, when the timing of the vaccination is less ideal (34). Additionally, college-age women are influenced by their peers and are more likely to get vaccinated if it is perceived as the social norm (44). Most of the studies conducted on patients’ attitudes toward the HPV vaccine focused in adolescents and adults but few explore the attitudes of 11- to 12-year-old girls who would ideally receive the vaccine. One small study did evaluate the attitudes of this population and found that 11- to 12-year-old girls are interested in information about vaccine efficacy and side effects, but a discussion of sexual health was less important to both the girls and their mothers (45).

### Physician Influence

The ACIP recommends HPV vaccine to be given to girls of ages between 11 and 12, at the same time as the tetanus–diphtheria–acellular pertussis (Tdap) vaccine and the four-valent meningococcal vaccine (46). However, parents and their daughters often rely on the physician to communicate these recommendations (45), and lack of physician recommendation is one of the most frequently cited reasons for not vaccinating preadolescent and adolescent women (38, 43). In a longitudinal study of 388 vaccine-eligible girls, only 37% received an HPV vaccine recommendation by the physician over the course of a year (47). The reason for this extraordinarily low rate of physician recommendation is only partially understood. Providers with low self-reported vaccination rates report delaying the vaccine in patients who they perceive to be at low risk for sexual activity. Thus, the ignorance and misconception from the health-care provider ultimately drive low vaccination initiation rates. This even goes so far that parents of these children report that their doctor supported or even suggested delaying the vaccine (40). In contrast, a positive physician recommendation has been shown to significantly increase intent to vaccinate (48). In a survey of over 17,000 parents of girls of ages 12–17, parents who had been counseled by a physician were 23 times more likely to have initiated vaccination and 14 times more likely to complete the series. In fact, differences in physician counseling practices may largely explain the differences in interstate vaccination rates (16).

The language used in communication also likely has a large influence on patients’ decision to vaccinate; approximately 25% of family practice physicians and pediatricians reported that they do not strongly endorse the vaccine themselves. Therefore, these physicians may not recommend vaccination to their patients at all, or if they do, the authors of the study suggest that they may appear ambivalent and, therefore, their recommendation is less likely to be pursued (49). Both pediatricians and family physicians often delay vaccination: they are both almost twice as likely to strongly recommend the vaccine to girls aged 13–15 vs. 11–12 (21). The sensitive nature of the vaccine, as it relates to sexual activity, also influences physician comfort discussing the vaccine. One study found that almost half of those surveyed felt that it was necessary to discuss sexuality before recommending the vaccine (21), and vaccine recommendation rates are lower in physicians who feel uncomfortable discussing sexuality (49).

## Interventions to Improve Delivery of the HPV Vaccine

There are two key themes to the barriers to HPV vaccination in the United States, which have been reviewed so far, and which much be addressed for any intervention to significantly impact uptake rates. First, misinformation and lack of education is prevalent among parents, physicians, and young women. Second, there has historically been a lack of access to care, either due to the cost of the vaccine for those with insurance or under- or uninsured status. The ACA created the “Prevention and Public Health Fund” which funds “Immunization Grants” provided by the CDC to programs, which are designed to improve vaccination rates (50). There are several different strategies, including education-based, systems-based, and region-based interventions that have been studied to determine, which might best address the current known barriers to vaccination.

### Educational Interventions

Many of the barriers to vaccination which have been described above highlight the need for education of all stakeholders: the parents, young women, and physicians. However, a recent systematic review of educational interventions to improve HPV vaccination rates concluded that the widespread implementation of educational interventions would be unsuccessful (51). This is likely due to the fact that different interventions are necessary to reach different communities, and each must be tailored to a specific audience. Gargano et al. (52) demonstrated the importance of understanding and targeting the audience in an intervention designed to increase adolescent awareness and interest in HPV vaccination. Their first step was a focus group to determine how best to reach the target community. They also incorporated the HPV education into the already-existent structure of the school through the use of the science teacher. By engaging the target community, they were able to significantly increase middle and high school students’ interest in vaccination through education (52). A study targeting Hispanic women first administered a survey to ensure the educational material was tailored toward the target population’s baseline knowledge and was able to demonstrate significant increase in intention to vaccinate (53). While these two studies were successful in increasing interest in vaccination and willingness to vaccinate, other attempts have been less successful. An online intervention called “MeFirst” incorporated college students’ baseline knowledge of HPV in order to design a tailored educational intervention; however, 3 months after the intervention, those randomized to the tailored education were no more likely to have initiated the vaccine series than those who had just read the CDC information face-sheet (54). Of note, few studies evaluate actual vaccination uptake outcomes, and most rely on changes in reported intention to vaccinate as a surrogate, which may over-estimate the impact of education on vaccination rates. Given the variability in results of these studies, it is unclear what impact educational interventions alone would ultimately have on HPV vaccination initiation and completion.

### Clinic-Based Interventions

Interventions with a systems-based approach have also been studied and are encouraged by the CDC as a mechanism to reach the Healthy People 2020 goal (10). One type of systems-based approach focuses upon intervention at the level of the practice and/or clinic. Standing orders, which authorize non-physician health-care personnel to administer a vaccine to an individual through a protocol approved by an authorized practitioner, are one evidenced-based method, which increases vaccination rates and is endorsed by the U.S. Preventive Services Task Force (55). A survey of young women attending a gynecology clinic found that standing orders for the HPV vaccine were generally acceptable to this population, particularly for the series completion (56).

Automatic reminders or recall-based interventions also increase vaccination rates: a systematic review of seven studies demonstrated that reminder systems for the parents or patients, whether that be through telephone calls, letters, text messages, or outreach visits, are consistently effective in improving HPV vaccination overall (57). These interventions are also relatively easy to implement and may be particularly helpful with improvement of vaccine completion rates (58). However, as with the educational interventions described above, this type of intervention may not work in all groups and may not work if used alone. In one study of mostly Hispanic and black parents attending a pediatric clinic in Texas, reminder phone calls resulted in improvements in rates of receipt of the second and third doses of the HPV vaccine, but only in Hispanic populations (59). These results further highlight the need to understand the community and culture when initiating and evaluating an intervention. Furthermore, the above data support a diverse and multifaceted approach to increasing vaccine uptake.

Another systems-based intervention encouraged by the CDC targets the physician and combines education, reminders, audits, and feedback to help address the physician contribution to low vaccination rates (10). In one cluster randomized controlled trial, clinics that received focused clinician education, electronic health record based alerts, and quarterly performance feedback for physicians had a modest, but statistically significant, increase in vaccine initiation compared to control clinics (58). Reminders for both parents and physicians can improve vaccination rates, and while alone, they will likely not get the United States to the Healthy People 2020 goal alone, they are a powerful adjunct to any vaccine promotion program.

### School-Based Interventions

School-based interventions are another type of systems-based approach that has been successful in several countries where there is already a framework for government-funded universal vaccination, such as Australia (8). While many believe that the concept of a school-based vaccine clinic is also feasible in the United States, some studies suggest that key stakeholders are skeptical of this approach. Focus groups in New Mexico were conducted with key stakeholders: parents of adolescent girls and boys, adolescent girls, middle school nurses, and middle school administrators and highlighted their concerns with this type of intervention. Overall, parents were uncertain about a middle school-based program, and school administrators felt that they lacked the implementation authority (60). However, other studies suggest that this type of intervention can be successful in the United States. School-based vaccine clinics seem particularly more feasible with the passage of the ACA and no-cost-sharing insurance coverage of the vaccine. In a cluster randomized controlled trial in Denver (CO, USA) in 2011, 16 schools were randomly assigned to a school-based vaccine clinic (*n* = 8) or a control. Clinics were held three times a year to accommodate the HPV vaccination schedule. Consent and insurance information was collected from the parents prior to vaccine administration. Compared to controls, children in the intervention schools were more likely to receive the Tdap vaccine, the meningococcal vaccine, and the HPV vaccine. The biggest increase was seen in HPV vaccination rates, where students were 70% more likely to receive the vaccine. One of the issues raised in this study was that less than half of the vaccine clinic costs were recuperated through insurance claims, although this may be related to their study population (over 40% of students were uninsured and were not charged for vaccine administration) (61). Regardless, this randomized controlled trial provides important evidence that school-based vaccine clinics are feasible and can be effective in the United States. Additional government funding or partnership with local agencies could help cover costs and, coupled with education, stands to have the biggest impact on vaccination rates. Furthermore, just as with the other types of interventions discussed, adjusting approaches to fit each community or school may ultimately be needed in order to gain widespread acceptance.

### State- and National-Level Interventions

Four of the six jurisdictions (Chicago, DC, Georgia, and Utah) which demonstrated improvements in HPV vaccine uptake from 2013 to 2014 had received funding through Prevention and Public Health Fund and had instituted a variety of interventions ranging from education to monetary support for vaccine programs (15). In addition to funding, state mandates may also have the potential to improve vaccination rates. Until recently, only two states had instituted a mandate for HPV vaccination. In 2008, Virginia passed a mandate for all girls entering the sixth grade to have at least one HPV vaccine, and in 2009, Washington, DC, USA, passed a similar mandate (15). Both included the ability to “opt-out” at parental discretion. These two states now have widely disparate rates of vaccine initiation, demonstrating the variability in the effectiveness of mandates. On the one hand, Washington, DC, USA, ranks number 2 for vaccine initiation in girls aged 13–17, and as previously mentioned was one of the only six jurisdictions to show improvement in vaccination rates from 2013 to 2014. On the other hand, Virginia still ranks 28th for vaccine initiation (see Figure 1) (15). Focus groups conducted in Virginia revealed that its public generally was not ready for the mandate: parents felt the government did not have the right to provide parental consent, they felt they did not have enough information about the new vaccine before the mandate was launched, they distrusted the motivations of Merck, and they wanted more education about the vaccine before having to make a decision (62).

State mandates may, therefore, be appropriate for some states, but not for others. In fact, a total of 24 states have previously tried to introduce a mandate into their legislatures, and only Virginia, DC, and Rhode Island have been successful in passing it into viable law (63). Rhode Island’s state mandate recently went into effect on August 1, 2015; it requires both girls and boys to have at least one dose of the vaccine to enter seventh grade this year, two doses to enter eighth grade by 2016, and three doses to enter ninth grade by 2017. In contrast to the DC and Virginia mandates, exceptions to vaccination are only made for physician-documented medical reasons or if it is against the parents’ religious beliefs (64). It remains to be seen if this mandate will be acceptable to the Rhode Island population, but the state currently ranks number 1 for vaccine uptake, which may reflect a positive attitude and predict acceptance of the vaccine in this population. Further research on the effectiveness of these mandates and other state-based interventions is needed to understand why particular interventions work in some regions and not others. It is unlikely that state mandates alone will help the United States reach its vaccination goal; lessons from Virginia’s mandate show us that education prior to mandate is the key.

### Finding the Right Intervention

Table 1 provides a matrix demonstrating the various proposed interventions and the barriers which they have the potential to address. An analysis of 21 HPV vaccination programs implemented in low- and middle-income countries found that tailoring the intervention to meet the community’s unique needs is an effective method of improving vaccination rates (24). This same principle can be applied in the United States, where it is unlikely that one intervention will achieve success in all regions and states. While education may help address physician, patient, and parent attitudes and beliefs, education alone has not been shown to improve the HPV vaccination rates enough to reach the 2020 goal. State mandates, if used alone, merely provide an incentive to vaccinate without addressing attitudes and lack of knowledge. Therefore, mandates can result in failure when education is lagging or not included. Reminder systems for physicians and patients, similarly, will not improve knowledge, but can help with vaccine completion rates. The ACA legislation has helped improve access but does not address knowledge or attitudes. Clearly, there is no intervention that will alone result in widespread uptake of the vaccine. From the available evidence, the optimal intervention would involve a school-based vaccine clinic combined with complementary parental and student education, addressing the majority of the barriers to vaccination. It is beyond the scope of this review to evaluate HPV vaccination uptake in males in the United States. However, similar interventions which increase vaccination rates in girls would likely work for both sexes if tailored toward improving knowledge, access, and acceptability in both populations. As vaccination coverage increases for both boys and girls, HPV vaccination will become more of a social norm, which will help perpetuate further vaccination of generations to come.

**Table 1:** **Addressing barriers through interventions: improving HPV vaccination rates**.

			Barriers to HPV vaccination					
			Parent/patient lack of knowledge	Physician bias	Regional differences	Follow-up (vaccination completion)	Access to care	Cost
Interventions	Individual level	Parent/patient educational interventions	X					
		Physician educational interventions		X				

	Clinic level	Parent/patient reminders and recalls				X		
		Physician reminders and feedback		X		X		

	School level	School-based vaccine clinics		X	X	X	X	
		School-based vaccine clinics with education	X	X	X	X	X	

	State/national level	State-based mandates		X			X	
		National no cost-sharing coverage (ACA)			X		X	X

*This matrix demonstrates potential HPV vaccination interventions at various levels, from individual to national, and the ways in which each intervention interacts with different barriers to vaccination. It is clear that there is not one intervention which alone can address all barriers, and a multipronged approach at the individual, clinic, state, and national levels will be necessary to reach the Healthy People 2020 goal of 80% vaccine completion in young women*.

*HPV, human papillomavirus*.

## Conclusion

The United States has a long way to go to reach the Healthy People 2020 goal of HPV vaccine coverage in over 80% of girls aged 13–17. The release of the nine-valent vaccine at the time of increasing vaccine uptake represents a possible tipping point in the fight against cervical cancer and could be the first step in the eradication of the HPV-related disease. An understanding of the attitudes and points of view of the various stakeholders is the key to designing interventions that are tailored to the needs of various communities. While education is the key for all, it will likely not be enough. Efficient and effective use of the electronic health record to remind physicians and parents about when vaccines are due is a proven option. Additionally, school-based vaccination methods hold the greatest promise here in the United States and have proven effective in other developed countries. The CDC encourages state and local public health departments to help lead HPV vaccination campaigns, to reach out and educate and motivate both parents and clinicians on HPV vaccination, and to incorporate HPV vaccination into each jurisdiction’s cancer control plans (10). State mandates are not enough. It is clear that a multifaceted approach is necessary to break down the barriers to HPV vaccination that is so prevalent in the United States.

## Author Contributions

Both authors certify that they contributed substantially to the conception, design, and analysis in this review, and both participated in drafting and revising the manuscript. Both authors approve of this review in its final form and take responsibility for its content.

## Conflict of Interest Statement

The authors declare that the research was conducted in the absence of any commercial or financial relationships that could be construed as a potential conflict of interest.
